# Implication of vaccination against dengue for Zika outbreak

**DOI:** 10.1038/srep35623

**Published:** 2016-10-24

**Authors:** Biao Tang, Yanni Xiao, Jianhong Wu

**Affiliations:** 1 School of Mathematics and Statistics, Xi'an Jiaotong University, Xi'an 710049, PR China; 2 Centre for Disease Modelling, York Institute for Health Research, York University, Toronto, ON, M3J 1P3, Canada

## Abstract

Zika virus co-circulates with dengue in tropical and sub-tropical regions. Cases of co-infection by dengue and Zika have been reported, the implication of this co-infection for an integrated intervention program for controlling both dengue and Zika must be addressed urgently. Here, we formulate a mathematical model to describe the transmission dynamics of co-infection of dengue and Zika with particular focus on the effects of Zika outbreak by vaccination against dengue among human hosts. Our analysis determines specific conditions under which vaccination against dengue can significantly increase the Zika outbreak peak, and speed up the Zika outbreak peak timing. Our results call for further study about the co-infection to direct an integrated control to balance the benefits for dengue control and the damages of Zika outbreak.

Dengue and Zika are both vector-borne diseases in tropical and sub-tropical regions with a common vector, dengue and Zika both belong to the family Flaviviridae and genus Flavivirus. Dengue is a prevalent disease being transmitted by the bite of a mosquito infected with one of the four serotypes^1^,^2^ while Zika is an emerging disease. Zika virus was first isolated in Uganda in 1947^3^, and there was an outbreak of Zika in Yap, Federated States of Micronesia^4^ in 2007, and in French Polynesia^5^ till 2013. By the end of January 2016, autochthonous circulation of Zika was reported in more than 20 countries or territories in South, Central, and North America and the Caribbean^6^,^7^,^8^,^9^,^10^,^11^,^12^, leading to the declaration of WHO that Zika virus is a global public health emergency^13^.

Recent clinical and experimental evidences support immunological cross-reactivity between dengue and Zika^14^,^15^,^16^,^17^. In particular, these evidences show that plasma to dengue was able to drive antibody-dependent enhancement of Zika infection. Co-circulation of multiple serotypes of dengue and dengue-Zika co-circulation have previously been reported in refs 18, 19, 20. In particular, co-infection of dengue and Zika were observed in two patients during the Zika outbreak in New Caledonia in 2014^18^, and in two patients during the Zika outbreak in Tuparetama of Brazil in 2015^19^. The co-circulation could be a potentially series public concern given that more than a third of the world’s population lives in countries where dengue is endemic^21^, with the dengue belt covering Central America, most of South America, sub-Saharan Africa, India, and South East Asia. Relevant to this co-infection is the development of vaccine products against dengue by *Sanofi Pasteur*, and the clinical trials by Butantan and Takeda. Thus, it is an important urgent issue for public health decision makers to know how dengue immunization program impacts Zika transmission when co-circulation becomes wide spread. Specially, under which conditions implemented dengue immunization control programs may boost the outbreak of Zika is no longer a thought-provoking issue. Developing a framework to address this issue through a mathematical model is the main objective of this study.

Much progress has been made for modelling dengue infection dynamics including the role of cross-reactive antibodies for the four different dengue serotypes as discussed in the review paper^22^. The dengue transmission dynamics becomes very complex because of the co-circulating serotypes in many endemic areas, and the absence of long-term cross-immunity^23^,^24^,^25^,^26^. In 1997, Feng *et al*.^27^ proposed a two-stain model with the vector population being subdivided into a susceptible class and two serotype-specific infectious classes and the host populations being described by the SIR-type model for each serotype. Esteva and Vargas^28^ considered a further model by including an explicit state for individuals who recovered from primary infections. Nuraini *et al*.^29^ and Sriprom *et al*.^30^ extended Esteva’s model by accounting for two separate symptomatic and asymptomatic compartments for secondary infections. A four-serotype model was considered in these papers^31^,^32^,^33^. Different from these previous studies, recently developed mathematical models have emphasized the evaluation of the impact of co-circulation of the four serotypes mainly among hosts^34^,^35^,^36^,^37^,^38^,^39^,^40^. In contrast to dengue, the epidemiology of Zika among humans remains poorly understood, despite some recent outbreaks of modelling activities^41^,^42^,^43^,^44^.

We should mention that mathematical models of co-infection of two infectious diseases among humans have been developed in many different settings^45^, including co-infection of HIV with TB^46^,^47^,^48^,^49^,^50^,^51^, HCV^52^,^53^, two strains of HIV^54^, HDV and HBV^55^, multi-strains of influenza^56^,^57^. To our best knowledge, our work here is the first attempt to develop a mathematical model to address the co-infection of dengue and Zika and its implication to Zika prevalence. Our purpose here is to propose a mathematical model of co-infection of dengue and Zika with particular focus on the potential impact and implication for Zika outbreak of vaccination against dengue in humans.

## Preliminaries

We stratify the total human population, *N*_*h*_(*t*), into:

*S*(*t*): the number of humans susceptible to both dengue and Zika at time *t*;

*I*_*d*_(*a, t*): the number of dengue-infected humans with infection age *a* at time *t*, who can also be infected by Zika virus and move to *I*_*dz*_(*a, b, t*);

*I*_*z*_(*b, t*): the number of Zika-infected humans with infection age *b* at time *t*, who can also be infected by dengue and move to *I*_*dz*_(*a, b, t*);

*I*_*dz*_(*a, b, t*): the number of dengue and Zika-infected humans with dengue infection age *a* and Zika infection age *b* at time *t*;

*R*_*d*_(*t*): the number of humans recovered from dengue at time *t*, who can also be infected by Zika and move to 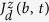;

*R*_*z*_(*t*): the number of humans recovered from Zika at time *t*, who can also be infected by dengue and move to 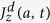;


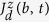
: the number of Zika-infected humans with Zika-infection age *b*, at time *t*, who are immune to dengue;


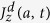
: the number of dengue-infected humans with dengue-infection age *a*, at time *t*, who are immune to Zika;

*R*_*dz*_(*t*): the number of humans recovered from dengue and Zika at time *t*, who can neither be infected by dengue nor Zika.

**Figure 1:**
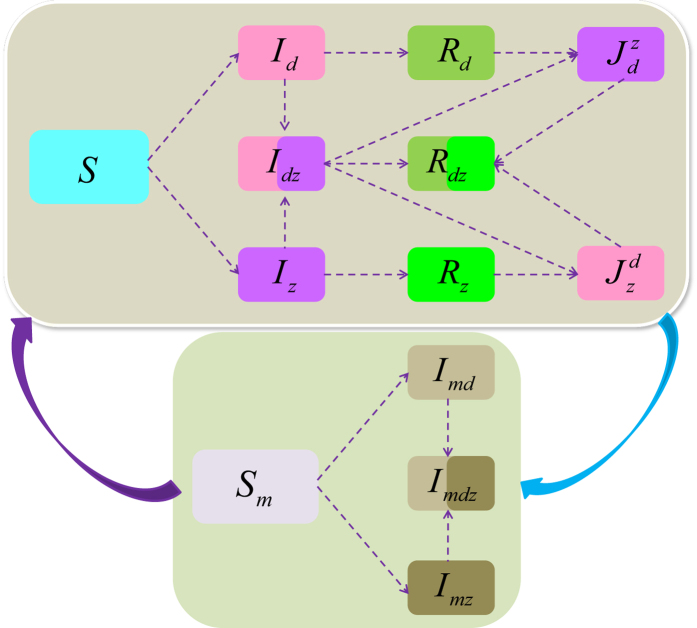
Transmission diagram.

Mosquito population *N*_*m*_ is divided into *S*_*m*_, *I*_*md*_, *I*_*mz*_, *I*_*mdz*_, representing the density of mosquitos who are susceptible, infected with dengue only, infected with Zika only, infected with both dengue and Zika. The transmission diagram of co-infection of dengue and Zika among humans and mosquitos is shown in Fig. 1.

**Figure 2:**
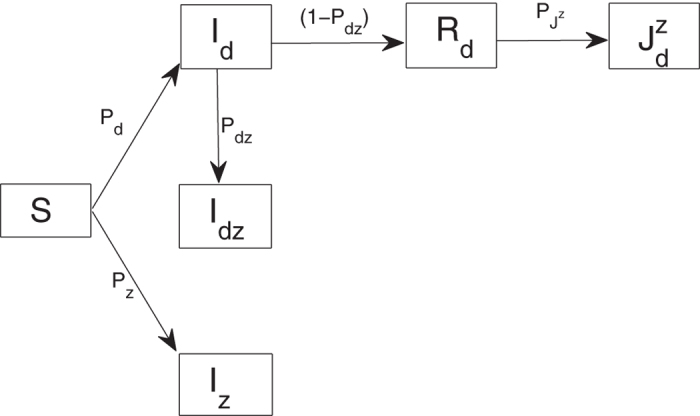
Sub-flow diagram of Zika infection among humans. Here, we assume that the susceptible humans (*S*) are infected by dengue with a ratio of *P*_*d*_ on average and by Zika with a ratio of *P*_*z*_ on average. We further assume that the class *I*_*d*_ will be infected with Zika at a ratio of *P*_*dz*_ while the other part will recover to *R*_*d*_. Moreover, we assume that the individuals in compartment *R*_*d*_ can be further infected with Zika at a ratio of 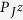.

We start with an intuitive view about the effects of vaccination against dengue among humans on the outbreak of Zika through a very simple static transmission model illustrated in Fig. 2. Here the susceptible humans (*S*) can be infected with Zika virus via three different routes, namely


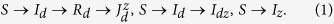


Let the initial number of susceptible humans (*S*) be *S*_0_. If we do not inoculate against dengue, then the final average number of humans infected with Zika virus through the above three routes (i.e. *I*_*z*_, *I*_*dz*_, 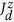) can be calculated as


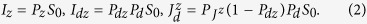


Therefore, the total number of humans infected with Zika virus should be


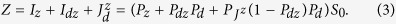


Now we assume that the coverage rate of dengue vaccine is *P*_*c*_ and the efficacy rate of dengue vaccine is *P*_0_. Then the effective coverage rate of dengue vaccine is *P*_*v*_ = *P*_0_*P*_*c*_. The portion of susceptible humans successfully inoculated with dengue vaccine will directly transfer to the compartment *R*_*d*_. Therefore, the final average numbers of *I*_*z*_, *I*_*dz*_ and 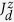 become


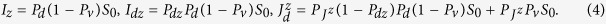


Then, the total number of humans infected with Zika virus after vaccination against dengue should be


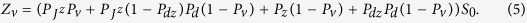


**Figure 3:**
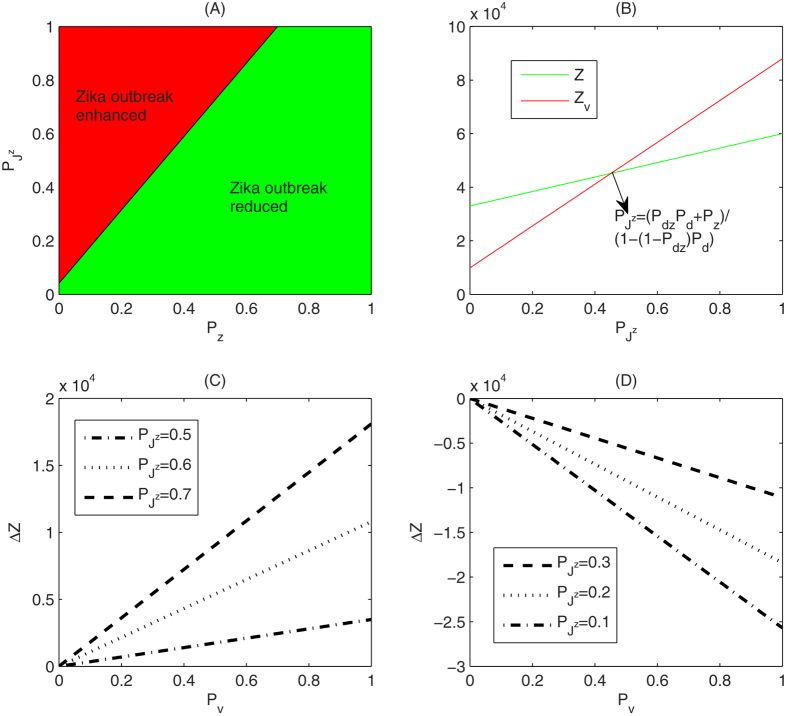
(**A**) Schematic scenarios which show that vaccination against dengue can increase the total number of Zike infections if the parameters *P*_*z*_ and 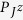 are located in the red region while it can decrease the total number of Zika infections in the green region; (**B**) The relationship of the total number of Zika infections to the ratio 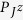 with or without vaccination against dengue. Here, *P*_*z*_ = 0.3 and *P*_*v*_ = 0.7; (**C**) The relationship of Δ*Z* to the effective coverage rate of dengue vaccine *P*_*v*_ while the parameters *P*_*z*_ and 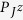 are chosen in the red region of (**A**) with *P*_*z*_ = 0.3; (**D**) The relationship of Δ*Z* to the effective coverage rate of dengue vaccine *P*_*v*_ while the parameters *P*_*z*_ and 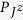 are chosen in the green region of (**A**) with *P*_*z*_ = 0.3. Other parameters in (**A–D**) are fixed as *P*_*d*_ = 0.3, *P*_*dz*_ = 0.1, *S*_0_ = 100000.

Comparing equation (2) with equation (4), we can see that with the implementation of vaccination of dengue the final numbers of *I*_*z*_ and *I*_*dz*_ decrease while the final number of 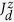 increases. To determine whether the total number of humans infected with Zika is increased or not, we let


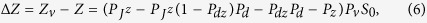


where 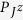 is the ratio at which the part of the susceptible humans inoculated with dengue vaccine are infected with Zika, 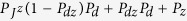 is the total ratio at which the susceptible humans are infected with Zika through the above mentioned three routes described in (1). It follows from equation (6) that if 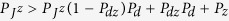 (i.e. 
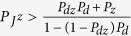
, as shown in the red region of Fig. 3(A)), then the higher ratio the susceptible humans are inoculated with dengue vaccine, the more the total number of humans are infected with Zika virus compared with the case without dengue vaccination, as shown in Fig. 3(B,C); if 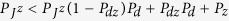 (i.e. 
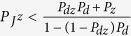
, as shown in the green region of Fig. 3(A)), inoculating dengue vaccine can decrease relatively the total number of humans infected with Zika virus, as shown in Fig. 3(B,D). This discussion, based on a static infection outcome analysis, suggests a likely scenario that, under certain conditions, vaccination against dengue can significantly boost the outbreak of Zika. Our analysis below is to theoretically and numerically examine these conditions with our proposed transmission dynamics model.

## Model formulation

We assume a SI-type model for dengue and Zika co-infection for the mosquito population. The model equations for mosquitos give


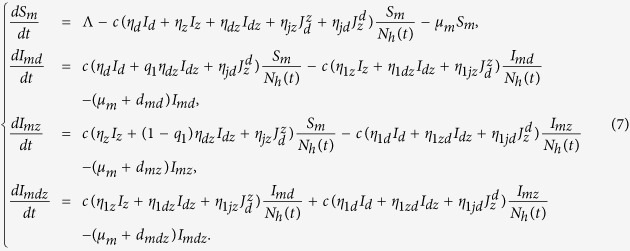


Here, Λ is the recruitment rate of mosquitos, and the definitions for other parameters are listed in Table 1. We assume SIR-type model for dengue and Zika co-infection in human population and formulate the following age-structured model to describe the dynamics of co-infection of dengue and Zika among humans:


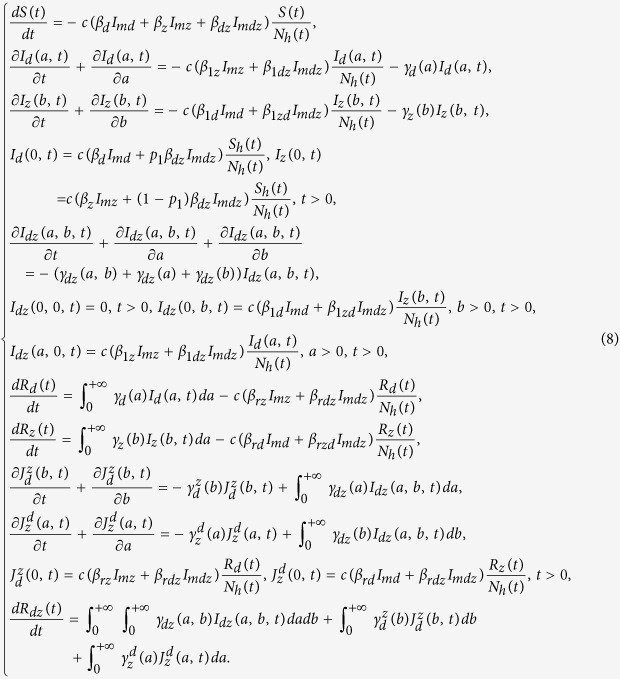


Here *γ*_*d*_(*a*) is the recover rate at which individuals in the compartment *I*_*d*_ with dengue-infection age *a* recover to the class *R*_*d*_, *γ*_*z*_(*b*) denotes the recover rate at which individuals in the class *I*_*z*_ with Zika-infection age *b* move to the compartment *R*_*z*_, 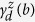 represents the recover rate at which individuals in the class 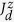 with Zika-infection age *b* recover to the compartment *R*_*dz*_, and 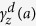 is the recover rate at which individuals in the class 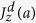 with dengue-infection age *a* move to compartment *I*_*dz*_, *γ*_*dz*_(*a, b*) denotes the recover rate at which individuals in the class *I*_*dz*_ with time-since-infection *a* for dengue and time-since-infection *b* for Zika recover to the compartment *R*_*dz*_ directly, *γ*_*dz*_(*a*) represents the recover rate at which individuals in the class *I*_*dz*_ transit to the compartment 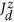 due to recovery of dengue, and *γ*_*dz*_(*b*) is the recover rate at which individuals in the class *I*_*dz*_ transit to the compartment 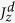 due to recovery of Zika. The definitions for other parameters independent of infection ages are given in Table 1. Here, the condition *I*_*dz*_(0, 0, *t*) = 0 means that the susceptible individuals can not be infected with dengue and Zika in the same time.

We assume that


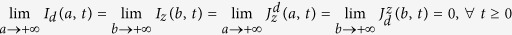


and


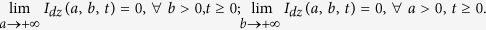


Define 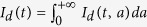, i.e. the total number of humans who are infected with dengue at time *t*, and can further be infected by Zika. Then, we have


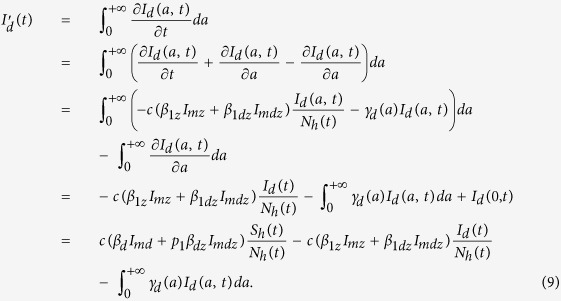


Further, if we assume that the recover rate *γ*_*d*_(*a*) is independent of dengue-infection age *a*, that is, *γ*_*d*_, we have 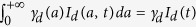. Then formula (9) yields


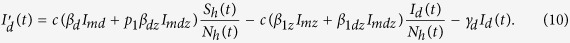


Similarly, if the recover rate *γ*_*z*_(*b*) is independent of Zika-infection age *b*, the total number of humans infected with Zika, given by 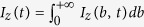, reads


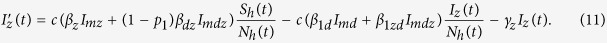


With similar calculation, we can get the derivative of the compartment *I*_*dz*_(*t*) as follows:


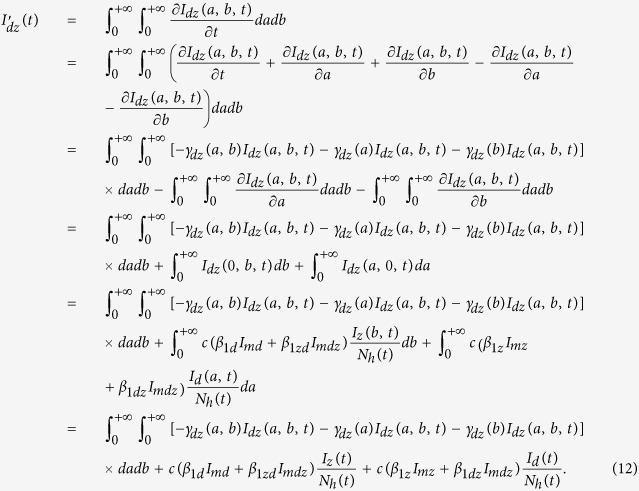


Also, when we assume that the recover rates *γ*_*dz*_(*a, b*), *γ*_*dz*_(*a*) and *γ*_*dz*_(*b*) are all constants, denoted by 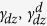 and 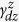, respectively, then formula (12) gives


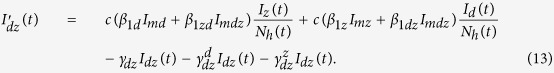


Moreover, define the total number of humans who are immune to dengue but infected with Zika as 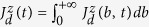 and the total number of humans who are immune to Zika but infected with dengue as 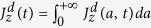. By assuming the recover rates 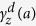 and 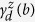 being independent of infection ages (i.e., 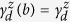 and 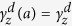), we easily obtain that


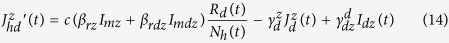


and


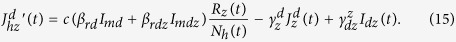


Based on the above assumptions and discussions, the double age-structured model is reduced to the following ODE model:


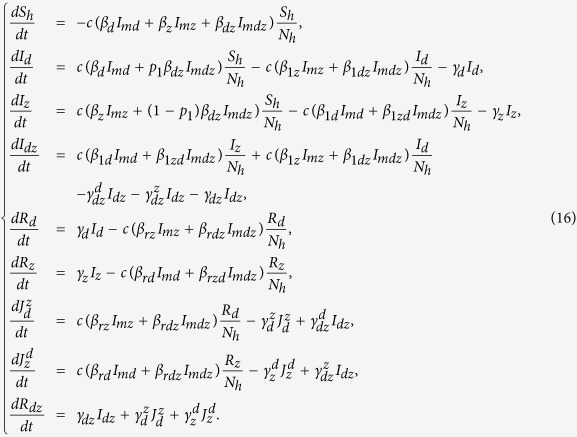


We call model (16) with model (7) as system S^*^. It follows from model (16) that the total number of humans *N*_*h*_(*t*) is a constant, denoted by *N*_*h*_. Let 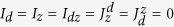 and *I*_*md*_ = *I*_*mz*_ = *I*_*mdz*_ = 0. Then we can show that system S^*^ has a disease-free equilibrium, which gives


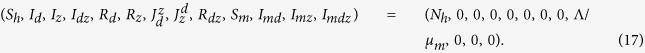


Using the next generation matrix introduced in papers^58^,^59^, we can calculate the basic reproduction number for system S^*^, denoted by *R*_0_ (see electronic supplementary information for details). This is the spectral radius of the next generation matrix and given by


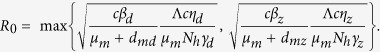


Here, 
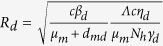
 and 
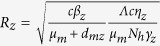
 are the basic reproduction numbers for the dengue-only model and Zika-only model, respectively. Consequently, when *R*_*z*_ > 1 (*R*_*d*_ > 1), then there is an outbreak of Zika (dengue) while the number of Zika (dengue) infections will directly decrease to zero if *R*_*z*_ < 1 (*R*_*d*_ < 1).

## Main Results

In this section, we carry out numerical simulations for the dynamic system S^*^ in order to examine effect of dengue vaccination on the outbreak of Zika. In our simulations, we vary three parameters *β*_*dz*_, *β*_*rz*_ and Λ, and fix all the other parameter values as follows:


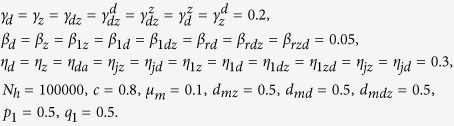


Let the initial values *IV*(0) for system S^*^ be given by


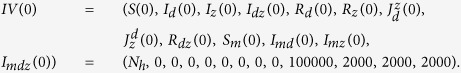


Let the effective coverage rate of vaccination against dengue among humans be *P*_*v*_. When inoculating dengue vaccine to humans at the outset of the outbreak of dengue and Zika, the initial conditions of model S^*^ become as 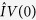 with 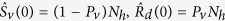 while other vector components remaining unchanged.

**Figure 4:**
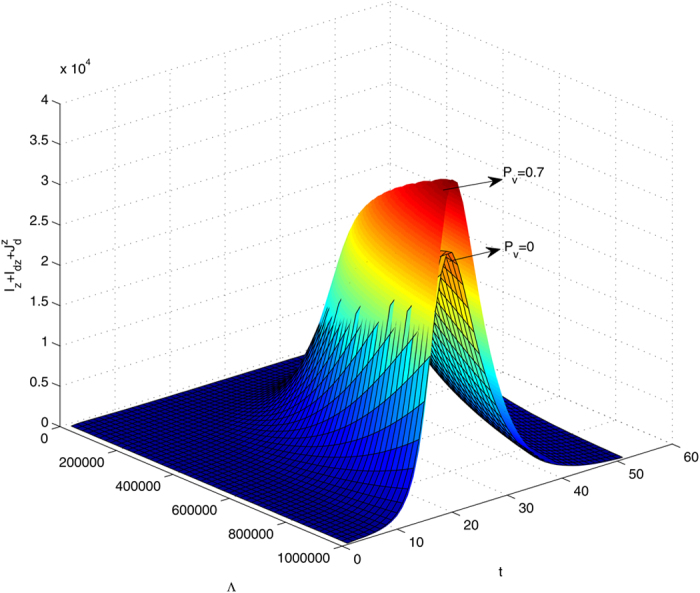
The value of 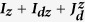 in time and with respect to the recruitment rate of mosquitos Λ being varied in the interval [10000, 1000000]. The mesh surface represents the solutions without inoculating dengue vaccine to susceptible humans while the other one are the solutions when the susceptible humans are inoculated with dengue vaccine at a ratio of 0.7. Parameters *β*_*dz*_ and *β*_*rz*_ are fixed as 0.18, 0.05, respectively.

**Figure 5:**
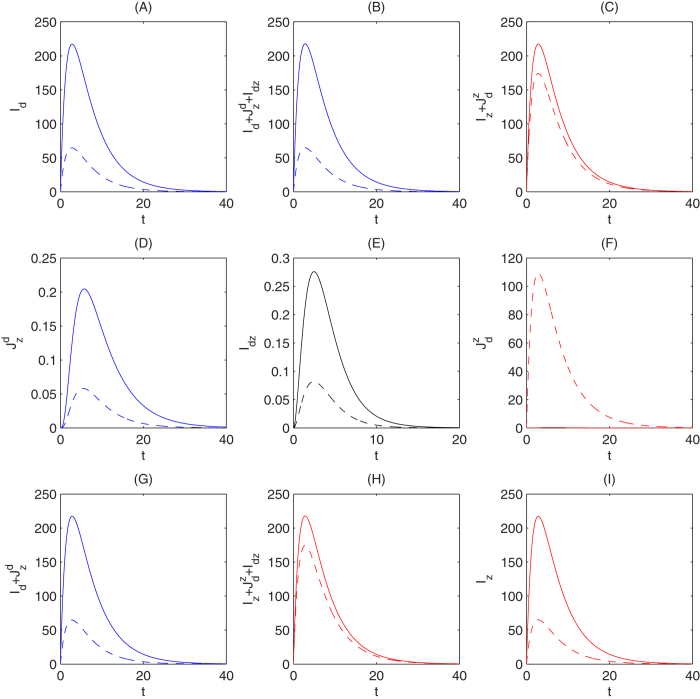
Solutions to system S^*^ with the solid curves being the solutions without vaccination and the dashed curves being the solutions with inoculating the dengue vaccine at the ratio of *P*_*v*_ = 0.7. Here, *β*_*dz*_ = 0.18, *β*_*rz*_ = 0.05, Λ = 10000.

**Figure 6:**
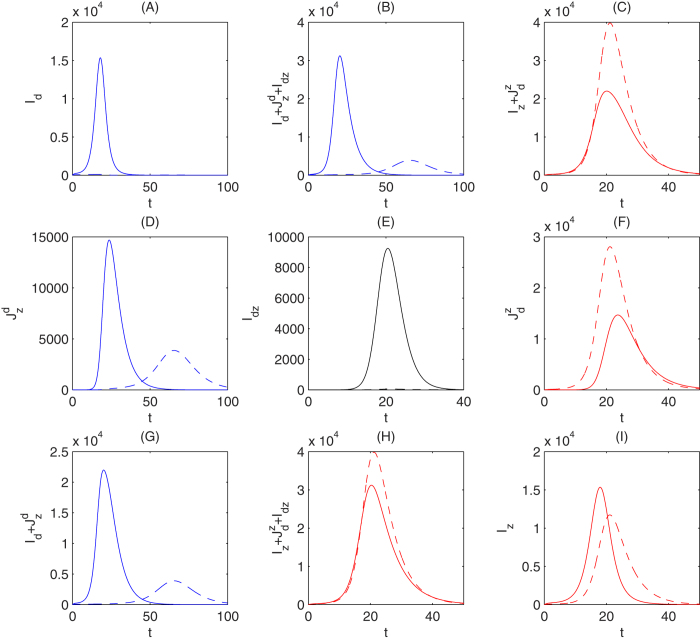
Solutions of model S^*^ with the solid lines being the solutions without vaccination and the dashed lines being the solutions after inoculating the dengue vaccine at the ratio of P_*v*_ = 0.7. Here we fixed Λ = 1000000 and all the other parameters as the same as those in Fig. 5.

We first simulate system S^*^ by fixing the parameters *β*_*dz*_ and *β*_*rz*_ as 0.18 and 0.05, respectively. We examine the variation of 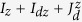 with parameter Λ with or without inoculating dengue vaccine, as shown in Fig. 4. As we can see, when the parameter Λ varies in the interval from 10000 to 1000000 vaccination against dengue can lead to two opposite results for the outbreak of Zika. That is, when Λ is relatively low, the effect of dengue vaccine on the outbreak of Zika is not noticeable. However, if Λ increases to relatively large, vaccination against dengue among humans will significantly boost the outbreak of Zika with a much higher outbreak peak compared with that without vaccination. The lower and upper bounds of this parameter value are determined from intensive numerical simulations to clearly illustrate these two opposite scenarios. In particular, we plot solutions to system S^*^ (shown in Figs 5 and 6) with Λ being fixed as 10000 and 1000000 (the lower and higher boundary value of the interval of Λ chosen in Fig. 4), respectively. Figures 5(H) and 6(H) demonstrate these two opposite situations: dengue vaccination results in the number of human infected with Zika either decline or increase. It follows from Figs 5 and 6 that vaccination against dengue among humans will always reduce the number of humans infected with dengue (including the compartments *I*_*d*_, 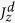, and *I*_*dz*_), and hence leads to a reduction in the total number of humans infected with dengue (i.e. 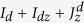). However, vaccination against dengue may increase the number of individuals in the compartment 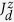. This explains the two opposite results about the effects of the dengue vaccination on the Zika outbreak. Note that when Λ = 1000000, with which vaccination against dengue can significantly boost the Zika outbreak, we can calculate that *R*_*d*_ = *R*_*z*_ = 2.82, within the range of basic reproduction numbers for dengue and Zika in the literatures^42^,^60^,^61^,^62^,^63^,^64^.

**Figure 7:**
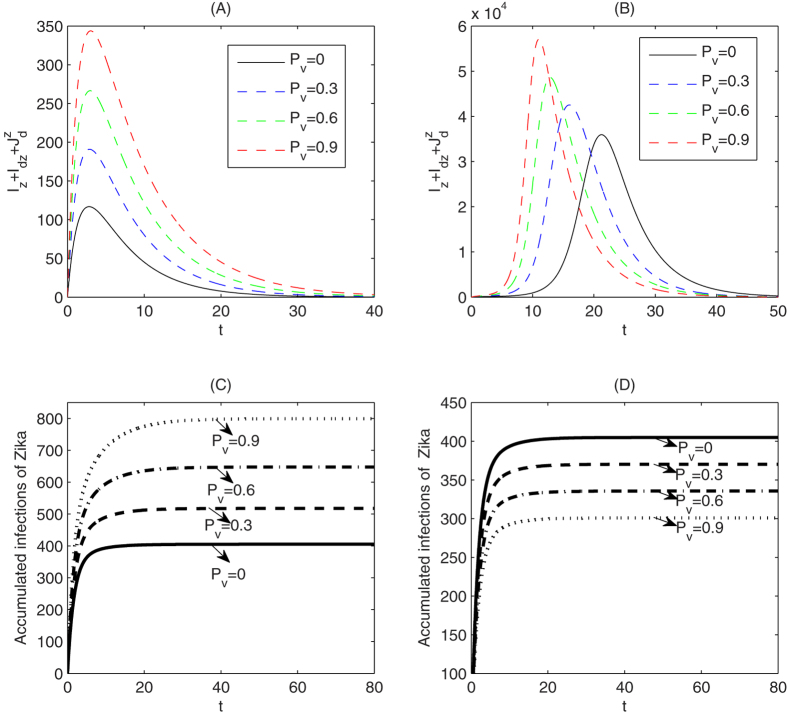
Solutions of 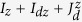 for (**A**) *β*_*dz*_ = 0.05, *β*_*rz*_ = 0.18, Λ = 10000 and (**B**) *β*_*dz*_ = 0.05, *β*_*rz*_ = 0.18, Λ = 1000000. The accumulated number of humans infected with Zika for (**C**) *β*_*dz*_ = 0.18, *β*_*rz*_ = 0.18, Λ = 10000 and (**D**) *β*_*dz*_ = 0.18, *β*_*rz*_ = 0.05, Λ = 10000.

Further, we examine the effects of the effective coverage rate *P*_*v*_ on the outbreak of Zika. Fix parameters *β*_*dz*_ = 0.05, *β*_*rz*_ = 0.18, Λ = 10000 and let the parameter *P*_*v*_ vary, Fig. 7(A) shows that a higher effective coverage rate of vaccination can result in a much higher peak of the outbreak of Zika. Moreover, if we choose Λ = 1000000, then we observe that with a higher rate of vaccination against dengue not only the peak of the outbreak of Zika can be significantly increased, but also the Zika outbreak peak much earlier, as shown in Fig. 7(B).

Considering the number of the accumulated Zika infections, we obtained two similar opposite results. Figure 7(C) shows that with a higher rate of vaccination against dengue the number of accumulated Zika infections will increase significantly, while Fig. 7(D) illustrates that vaccination against dengue may reduce the number of the accumulated Zika infections. In Fig. 7(D) we assumed that *β*_*rz*_ = *β*_*z*_ = 0.05 while in Fig. 7(C) we assumed that 0.18 = *β*_*rz*_ > *β*_*z*_ = 0.05 based on the emerging clinical evidence of enhancement^14^,^15^,^16^,^17^. Comparisons between these scenarios clearly indicate, under the conditions reflected by the parameter values, that dengue vaccination may indeed lead to significant increase in Zika infections.

## Conclusion and Discussion

There are increasing evidence of co-infection of dengue and Zika. Due to similar transmission routes with the same host species, some intervention strategies such as vector control are effective for curbing both dengue and Zika. However, other interventions such as vaccination against one virus may be harmful to the control of another, specially when enhancement occurs to favor the spread of the virus not covered with vaccine. Our study examined the implication of this enhancement for Zika outbreaks when vaccination against dengue in humans is applied. We initially formulated a very simple static transmission model to give an intuitive illustration that vaccination against dengue among humans may significantly boost Zika transmission among the population.

In order to theoretically verify this illustration, we then proposed a dynamic model to describe the dynamics of co-infection of dengue and Zika. More specifically, we developed a novel model with double age-structures for dengue and Zika, extending the general age-structure model^65^,^66^,^67^ by incorporating compartments with specific dengue-infection and Zika-infection age. Under certain stage-specific homogenetical assumptions about the virus dynamics characteristics, we simplified our double age-structured model to an ODE model, for which the basic reproduction number can be calculated.

We also numerically investigated the dynamics of model S^*^ and obtained some observations which are in agreement with the conclusions from the analysis of our static transmission model in Section 2. Figure 4 shows that vaccination against dengue among humans may result in the total number of humans infected with Zika virus decline or increase, depending on the parameter Λ, the recruitment rate of mosquitos. In particular, it significantly enlarges the peak of the outbreak of Zika when Λ is relatively large. It follows from Figs 5 and 6 that this enlarged outbreak of Zika by vaccination against dengue is due to multiple factors. Vaccination against dengue can reduce the numbers of *I*_*z*_ and *I*_*dz*_ while it always increases the number of 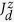. Thus the balance of increase in the number of 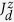 and decrease in the number of *I*_*z*_ and *I*_*dz*_ determines whether the total number of infected with Zika increase or not. Further, we observed that a higher rate of vaccination against dengue can also results in a higher and earlier peak of the outbreak of Zika, as shown in Fig. 7(A,B). Comparing Fig. 7(B) with Fig. 7(A), we observe that the conclusion that vaccination against dengue can boost Zika outbreak remains true for a wide range of mosquito index values (when the recruitment rate of mosquito decreases from 1000000 to 10000). This conclusion is also shown in Fig. S2 (electronic supplementary information) when the mosquito mortality rate *μ*_*m*_ varies. Comparison between Fig. 7(B) and Fig. 7(A) however also shows that reducing the mosquito indices can significantly decrease the magnitude of Zika outbreak as the number of Zika cases at the peak time can be reduced substantially. Therefore, given the simultaneous impact on both dengue and Zika outbreaks, vector control should be always implemented regardless of the availability of vaccine. Figure 7(C,D) further confirm that the accumulated Zika infections may be greater for a greater rate of vaccination of dengue vaccine to human. Sensitive analyses show that parameters *β*_*z*_, *β*_*dz*_, Λ and *μ*_*m*_ can significantly affect the outbreak of Zika, in terms of both the accumulated Zika infections and the daily number of Zika infections (see electronic supplementary information for details).

Most existing studies on the multi-serotype models of vector-host transmission of dengue focus on the importance of subsequent infections with different dengue serotypes. It was assumed that the patients can be subsequently infected by another serotypes after recovering from one serotype. In our consideration of dengue-Zika co-infection, we extended these models by adding a new compartment of humans as well as mosquitos infected by both of Zika and dengue simultaneously. From our numerical analysis, the parameter *β*_*dz*_ (i.e. the transmission rate of the compartment of mosquitos infected with dengue and Zika to susceptible humans), which is related to the newly added compartment *I*_*mdz*_, can have important influences on the dynamics of the co-infection model. For the models of co-infection of HIV with TB and HCV, a SI-type model is usually assumed as the basic model for each disease. In comparison with these models, our model with SIR-type for humans is different to handle the asymmetric vector-host interaction as discussed in ref. 27, and to allow recovered (or vaccinated) individuals from one virus to have higher risk of infection by another. Our analysis indicates that with a big recruitment rate of mosquitos Λ vaccination against dengue among humans can significantly boost the Zika outbreak (as shown in Fig. 6(H)), and cause the Zika outbreak peak coming early with a bigger mosquito to humans transmission rate *β*_*rz*_ and lower *β*_*dz*_ (as shown in Fig. 7(B)). It is important to note that a safe, effective and affordable dengue vaccine against the four strains offers an important tool to reach the WHO goal of reducing dengue morbidity by at least 25% and mortality by at least 50% by 2020^68^. The first dengue vaccine, Dengvaxiar(CYD-TDV) (developed by *Sanofi Pasteur*), was licensed in Mexico in 2015^69^; and two dengue vaccine candidates (developed by Butantan and Takeda) entered the Phase III trails in early 2016^70^,^71^,^72^. Our study should not serve as a discouragement to the development of these dengue vaccine products, but rather we determine conditions under which dengue vaccination can contribute to the prevention and control of dengue without inducing significant increase in Zika infection.

Most published works focus on the benefits of the control strategies (such as treatments for only one or both diseases) to both diseases involved in the co-infection. For example, Derouich and Boutayeb^73^ considered a model of two subsequent infections of dengue at separate time intervals with continuous vaccination. They concluded that vaccination can be a control strategy for dengue. However, with consideration of co-infection and the current development of dengue vaccine, our results suggest that additional study on co-infection is urgently and critically needed.

## Additional Information

**How to cite this article**: Tang, B. *et al*. Implication of vaccination against dengue for Zika outbreak. *Sci. Rep.* **6**, 35623; doi: 10.1038/srep35623 (2016).

## Supplementary Material

Supplementary Information

## Figures and Tables

**Table 1:** Definitions of the parameters.

Parameters	Definitions
*c*	Biting rate bites (per mosquito per day) (day^−1^)
*β*_*d*_	Mosquito (with dengue) -to-human transmission probability
*β*_*z*_	Mosquito (with Zika) -to-human transmission probability
*β*_*dz*_	Mosquito (with both) -to-human transmission probability
*β*_1*d*_	Mosquito (with dengue) -to-human (with Zika) transmission probability
*β*_1*z*_	Mosquito (with Zika) -to-human (with dengue) transmission probability
*β*_1*dz*_	Mosquito (with both) -to-human (with dengue) transmission probability
*β*_1*zd*_	Mosquito (with both) -to-human (with Zika) transmission probability
*β*_*rd*_	Mosquito (with dengue) -to-human (with Zika immune) transmission probability
*β*_*rz*_	Mosquito (with Zika) -to-human (with dengue immune) transmission probability
*β*_*rdz*_	Mosquito (with both virus) -to-human (with dengue immune) transmission probability
*β*_*rzd*_	Mosquito (with both virus) -to-human (with Zika immune) transmission probability
*η*_*d*_	Human (with dengue) -to-mosquito transmission probability
*η*_*z*_	Human (with Zika) -to-mosquito transmission probability
*η*_*dz*_	Human(with both) -to-mosquito transmission probability
*η*_*jd*_	Human (with dengue infection but Zika immune) -to-mosquito transmission probability
*η*_*jz*_	Human (with Zika infection but dengue immune) -to-mosquito transmission probability
*η*_1*d*_	Human (with dengue infection) -to-mosquito (with Zika infection) transmission probability
*η*_1*z*_	Human (with Zika infection) -to-mosquito (with dengue infection) transmission probability
*η*_1*dz*_	Human (with both virus) -to-mosquito (with dengue infection) transmission probability
*η*_1*zd*_	Human (with both virus) -to-mosquito (with Zika infection) transmission probability
*μ*_*m*_	Mosquito mortality rate (day^−1^)
*d*_*m*_	Mosquito disease -related mortality rate (day^−1^)
