# Supplementary material for: Implication of vaccination against dengue for Zika outbreak
Source: Sci Rep. 2016 Oct 24;6:35623. doi: 10.1038/srep35623 (PMC5075941; doi:10.1038/srep35623)
Supplement: Supplementary Information [file srep35623-s1.pdf]

Electronic supplementary information: Implication of vaccination against dengue for Zika outbreak

Biao Tang[a,b], Yanni Xiao[a 1], Jianhong Wu[b]

a School of Mathematics and Statistics, Xi'an Jiaotong University, Xi'an 710049, PR China

b Centre for Disease Modelling, York Institute for Health Research, York University, Toronto, ON, M3J 1P3, Canada.

Calculation for R_0

To calculate the basic reproduction number of model S^*, we consider the following equations

$$\left\{\begin{array}{lcl}
\frac{dI_d}{dt} &=& c(\beta_d I_{md} + p_1\beta_{dz}I_{mdz})\frac{S_h}{N_h} - c(\beta_{1z}I_{mz} + \beta_{1dz}I_{mdz})\frac{I_d}{N_h} - \gamma_d I_d,\\
\frac{dI_z}{dt} &=& c(\beta_z I_{mz} + (1-p_1)\beta_{dz}I_{mdz})\frac{S_h}{N_h} - c(\beta_{1d}I_{md} + \beta_{1zd}I_{mdz})\frac{I_z}{N_h} - \gamma_z I_z,\\
\frac{dI_{dz}}{dt} &=& c(\beta_{1d}I_{md} + \beta_{1zd}I_{mdz})\frac{I_z}{N_h} + c(\beta_{1z}I_{mz} + \beta_{1dz}I_{mdz})\frac{I_d}{N_h},\\
&& -\gamma^d_{dz}I_{dz} - \gamma^z_{dz}I_{dz} - \gamma_{dz}I_{dz},\\
&&\\
\frac{dJ^z_d}{dt} &=& c(\beta_{rz}I_{mz} + \beta_{rdz}I_{mdz})\frac{R_d}{N_h} - \gamma^z_d J^z_d + \gamma^d_{dz}I_{dz},\\
\frac{dJ^d_z}{dt} &=& c(\beta_{rd}I_{md} + \beta_{rdz}I_{mdz})\frac{R_z}{N_h} - \gamma^d_z J^d_z + \gamma^z_{dz}I_{dz},\\
&&\\
\frac{dI_{md}}{dt} &=& c(\eta_d I_d + q_1\eta_{dz}I_{dz} + \eta_{jd}J^d_z)\frac{S_m}{N_h} - c(\eta_{1z}I_z + \eta_{1dz}I_{dz} + \eta_{1jz}J^z_d)\frac{I_{md}}{N_h},\\
&& -(\mu_m + d_{md})I_{md}\\
\frac{dI_{mz}}{dt} &=& c(\eta_z I_z + (1-q_1)\eta_{dz}I_{dz} + \eta_{jz}J^z_d)\frac{S_m}{N_h} - c(\eta_{1d}I_d + \eta_{1zd}I_{dz} + \eta_{1jd}J^d_z)\frac{I_{mz}}{N_h}\\
&& -(\mu_m + d_{mz})I_{mz}\\
\frac{dI_{mdz}}{dt} &=& c(\eta_{1z}I_z + \eta_{1dz}I_{dz} + \eta_{1jz}J^z_d)\frac{I_{md}}{N_h} + c(\eta_{1d}I_d + \eta_{1zd}I_{dz} + \eta_{1jd}J^d_z)\frac{I_{mz}}{N_h}.\\
&& -(\mu_m + d_{mdz})I_{mdz}
\end{array}\right. \tag{1}$$

Let

$$\mathcal{F} = \begin{pmatrix}
c(\beta_d I_{md} + p_1\beta_{dz}I_{mdz})\frac{S_h}{N_h}\\
c(\beta_z I_{mz} + (1-p_1)\beta_{dz}I_{mdz})\frac{S_h}{N_h}\\
c(\beta_{1d}I_{md} + \beta_{1zd}I_{mdz})\frac{I_z}{N_h} + c(\beta_{1z}I_{mz} + \beta_{1dz}I_{mdz})\frac{I_d}{N_h}\\
c(\beta_{rz}I_{mz} + \beta_{rdz}I_{mdz})\frac{R_d}{N_h}\\
c(\beta_{rd}I_{md} + \beta_{rdz}I_{mdz})\frac{R_z}{N_h}\\
c(\eta_d I_d + q_1\eta_{dz}I_{dz} + \eta_{jd}J^d_z)\frac{S_m}{N_h}\\
c(\eta_z I_z + (1-q_1)\eta_{dz}I_{dz} + \eta_{jz}J^z_d)\frac{S_m}{N_h}\\
c(\eta_{1z}I_z + \eta_{1dz}I_{dz} + \eta_{1jz}J^z_d)\frac{I_{md}}{N_h} + c(\eta_{1d}I_d + \eta_{1zd}I_{dz} + \eta_{1jd}J^d_z)\frac{I_{mz}}{N_h}
\end{pmatrix}$$

[1]Corresponding author. E-mail: yxiao@mail.xjtu.edu.cn, Tel: +86 29 82663156, Fax:+86 29 82668551

and

$$\mathcal{V} = \begin{pmatrix} c(\beta_{1z} I_{mz} + \beta_{1dz} I_{mdz}) \frac{I_d}{N_h} + \gamma_d I_d \\ c(\beta_{1d} I_{md} + \beta_{1zd} I_{mdz}) \frac{I_z}{N_h} + \gamma_z I_z \\ \gamma_{dz}^d I_{dz} + \gamma_{dz}^z I_{dz} + \gamma_{dz} I_{dz} \\ \gamma_d^z J_d^z - \gamma_{dz}^d I_{dz} \\ \gamma_z^d J_z^d - \gamma_{dz}^z I_{dz} \\ c(\eta_{1z} I_z + \eta_{1dz} I_{dz} + \eta_{1jz} J_d^z) \frac{I_{md}}{N_h} + (\mu_m + d_{md}) I_{md} \\ c(\eta_{1d} I_d + \eta_{1zd} I_{dz} + \eta_{1jd} J_z^d) \frac{I_{mz}}{N_h} + (\mu_m + d_{mz}) I_{mz} \\ (\mu_m + d_{mdz}) I_{mdz} \end{pmatrix}$$

Then, there are

$$V = \begin{pmatrix} \gamma_d & 0 & 0 & 0 & 0 & 0 & 0 & 0 \\ 0 & \gamma_z & 0 & 0 & 0 & 0 & 0 & 0 \\ 0 & 0 & \gamma_{dz}^d + \gamma_{dz}^z + \gamma_{dz} & 0 & 0 & 0 & 0 & 0 \\ 0 & 0 & -\gamma_{dz}^d & \gamma_d^z & 0 & 0 & 0 & 0 \\ 0 & 0 & -\gamma_{dz}^z & 0 & \gamma_z^d & 0 & 0 & 0 \\ 0 & 0 & 0 & 0 & 0 & \mu_m + d_{md} & 0 & 0 \\ 0 & 0 & 0 & 0 & 0 & 0 & \mu_m + d_{mz} & 0 \\ 0 & 0 & 0 & 0 & 0 & 0 & 0 & \mu_m + d_{mdz} \end{pmatrix}$$

$$F = \begin{pmatrix} 0 & 0 & 0 & 0 & 0 & c\beta_d & 0 & p_1 c\beta_{dz} \\ 0 & 0 & 0 & 0 & 0 & 0 & c\beta_z & (1-p_1) c\beta_{dz} \\ 0 & 0 & 0 & 0 & 0 & 0 & 0 & 0 \\ 0 & 0 & 0 & 0 & 0 & 0 & 0 & 0 \\ 0 & 0 & 0 & 0 & 0 & 0 & 0 & 0 \\ c\frac{\eta_d \Lambda}{\mu_m N_h} & 0 & cq_1 \frac{\eta_{dz} \Lambda}{\mu_m N_h} & 0 & c\frac{\eta_{jd} \Lambda}{\mu_m N_h} & 0 & 0 & 0 \\ 0 & c\frac{\eta_z \Lambda}{\mu_m N_h} & c(1-q_1) \frac{\eta_{dz} \Lambda}{\mu_m N_h} & c\frac{\eta_{jz} \Lambda}{\mu_m N_h} & 0 & 0 & 0 & 0 \\ 0 & 0 & 0 & 0 & 0 & 0 & 0 & 0 \end{pmatrix}$$

Further, we can get

$$V^{-1}=\begin{pmatrix}
\frac{1}{\gamma_d} & 0 & 0 & 0 & 0 & 0 & 0 & 0\\
0 & \frac{1}{\gamma_z} & 0 & 0 & 0 & 0 & 0 & 0\\
0 & 0 & \frac{1}{\gamma_{dz}^d+\gamma_{dz}^z+\gamma_{dz}} & 0 & 0 & 0 & 0 & 0\\
0 & 0 & \frac{\gamma_{dz}^d}{\gamma_d^z(\gamma_{dz}^d+\gamma_{dz}^z+\gamma_{dz})} & \frac{1}{\gamma_d^z} & 0 & 0 & 0 & 0\\
0 & 0 & \frac{\gamma_{dz}^z}{\gamma_z^d(\gamma_{dz}^d+\gamma_{dz}^z+\gamma_{dz})} & 0 & \frac{1}{\gamma_z^d} & 0 & 0 & 0\\
0 & 0 & 0 & 0 & 0 & \frac{1}{\mu_m+d_{md}} & 0 & 0\\
0 & 0 & 0 & 0 & 0 & 0 & \frac{1}{\mu_m+d_{mz}} & 0\\
0 & 0 & 0 & 0 & 0 & 0 & 0 & \frac{1}{\mu_m+d_{mdz}}
\end{pmatrix}$$

and

$$FV^{-1}=\begin{pmatrix}
0 & 0 & 0 & 0 & 0 & a_{16} & 0 & a_{18}\\
0 & 0 & 0 & 0 & 0 & 0 & a_{27} & a_{28}\\
0 & 0 & 0 & 0 & 0 & 0 & 0 & 0\\
0 & 0 & 0 & 0 & 0 & 0 & 0 & 0\\
0 & 0 & 0 & 0 & 0 & 0 & 0 & 0\\
a_{61} & 0 & a_{63} & 0 & a_{65} & 0 & 0 & 0\\
0 & a_{72} & a_{73} & a_{74} & 0 & 0 & 0 & 0\\
0 & 0 & 0 & 0 & 0 & 0 & 0 & 0
\end{pmatrix}$$

where

$$\begin{aligned}
&a_{16}=\frac{c\beta_d}{\mu_m+d_{md}}, a_{18}=\frac{p_1c\beta_{dz}}{\mu_m+d_{mdz}}, a_{27}=\frac{c\beta_z}{\mu_m+d_{mz}}, a_{28}=\frac{(1-p_1)c\beta_{dz}}{\mu_m+d_{mdz}},\\
&a_{61}=\frac{\Lambda c\eta_d}{\mu_m N_h\gamma_d}, a_{63}=\frac{\Lambda}{\mu_m N_h(\gamma_{dz}^d+\gamma_{dz}^z+\gamma_{dz})}\left(c\eta_{jd}\frac{\gamma_{dz}^z}{\gamma_z^d}+cq_1\eta_{dz}\right), a_{65}=\frac{\Lambda c\eta_{jd}}{\mu_m N_h\gamma_z^d},\\
&a_{72}=\frac{\Lambda c\eta_z}{\mu_m N_h\gamma_z}, a_{73}=\frac{\Lambda}{\mu_m N_h(\gamma_{dz}^d+\gamma_{dz}^z+\gamma_{dz})}\left(c\eta_{jz}\frac{\gamma_{dz}^d}{\gamma_d^z}+c(1-q_1)\eta_{dz}\right), a_{74}=\frac{\Lambda c\eta_{jz}}{\mu_m N_h\gamma_d^z}.
\end{aligned}$$

Let $|\lambda I - FV^{-1}| = 0$, we have that

$$\begin{vmatrix} \lambda & 0 & 0 & 0 & 0 & -a_{16} & 0 & -a_{18} \\ 0 & \lambda & 0 & 0 & 0 & 0 & -a_{27} & -a_{28} \\ 0 & 0 & \lambda & 0 & 0 & 0 & 0 & 0 \\ 0 & 0 & 0 & \lambda & 0 & 0 & 0 & 0 \\ 0 & 0 & 0 & 0 & \lambda & 0 & 0 & 0 \\ -a_{61} & 0 & -a_{63} & 0 & -a_{65} & \lambda & 0 & 0 \\ 0 & -a_{72} & -a_{73} & -a_{74} & 0 & 0 & \lambda & 0 \\ 0 & 0 & 0 & 0 & 0 & 0 & 0 & \lambda \end{vmatrix} =$$

$$\lambda^4 \begin{vmatrix} \lambda & 0 & -a_{16} & 0 \\ 0 & \lambda & 0 & -a_{27} \\ -a_{61} & 0 & \lambda & 0 \\ 0 & -a_{72} & 0 & \lambda \end{vmatrix} = \lambda^4(\lambda^2 - a_{16}a_{61})(\lambda^2 - a_{27}a_{72}) = 0.$$

Thus, the basic reproduction number can be calculated as

$$R_0 = \max\left\{ \sqrt{\frac{c\beta_d}{\mu_m + d_{md}} \frac{\Lambda c \eta_d}{\mu_m N_h \gamma_d}}, \sqrt{\frac{c\beta_z}{\mu_m + d_{mz}} \frac{\Lambda c \eta_z}{\mu_m N_h \gamma_z}} \right\}.$$

Sensitive analysis

We explored the parameter space by performing an uncertainty analysis using a Latin hypercube sampling method. Through evaluating the partial rank correlation coefficients (PRCCs), we performed the sensitive analysis of the accumulated Zika infections and the daily number of Zika infections $(I_z + I_{dz} + J_d^z)$ with respect to the parameters β_z, β_{dz}, Λ and μ_m over time, as shown in Fig.S1. In the absence of data to inform distribution functions, we chose the uniform distribution for the control parameters β_z and β_{dz} with a range of $(0.001, 0.1)$ [1] and μ_m with a range of $(0.05, 0.1)$ [2]. It is reasonable to assume a constant number of mosquitoes per person and we assumed the constant ratio to be 3 [3]. Therefore, the total number of mosquitos is approximately 300000, as we have chosen $N_h = 100000$. Note that, if we do not consider the disease-related mortality rate for mosquitos, the total number of mosquitos approaches Λ/μ_m. Thus, we can let Λ obey the uniform distribution of the interval (15000,30000). The parameter P_v is fixed as 0.6 and the other parameter values are fixed as those in Fig.7(C).

Corresponding to the LHS matrix and scheme defined by varying all input parameters, Fig.S1(A-B) gives 1000 outputs of the accumulated Zika infections and the number of Zika infections, respectively. Fig.S1(C-D) shows the PRCCs over time with respect to the accumulated Zika infections and the daily number of Zika infections, respectively. As we can see in Fig.S1(C-D), all the input parameters can have lager PRCCs. As the parameter β_z changes, it is always correlated to large PRCCs over time. As the parameter Λ (μ_m) changes, it is initially positively (negatively) correlated to small PRCCs, and then significantly correlated to large PRCCs. However, the parameter β_{dz} is initially

positively correlated to a large PRCCs, and then correlated to a decline trend.

Simulation of the impact of the mosquito mortality rate on the outbreak of Zika

By varying the mosquito mortality rate and the rate of vaccination against dengue, while fixing the other parameter values as the same as those in Fig.7(A), we plot the solutions of $I_z + I_{dz} + J_d^z$, as shown in Fig.S2. It shows that vaccination against dengue can still boost the Zika outbreak with $\mu_m = 0.1$ and $\mu_m = 0.3$. However, the Zika cases at the peak time also decreases substantially with increasing the mosquito mortality rate. Vector control remains an effective option for managing both dengue and Zika outbreaks.

References

[1] Gao D. Z., et al. Prevention and control of Zika fever as a mosquito-borne and sexually transmitted disease. arXiv:1604.04008 (2016).

[2] Yakob, L. & Clements, A.C. A mathematical model of Chikungunya dynamics and control: the major epidemic on Reunion Island. PloS ONE 8(3):e57448 (2013).

[3] Chowell, G., et al. Estimation of the reproduction number of dengue fever from spatial epidemic data. Math. Biosci. 208, 571-589 (2007).

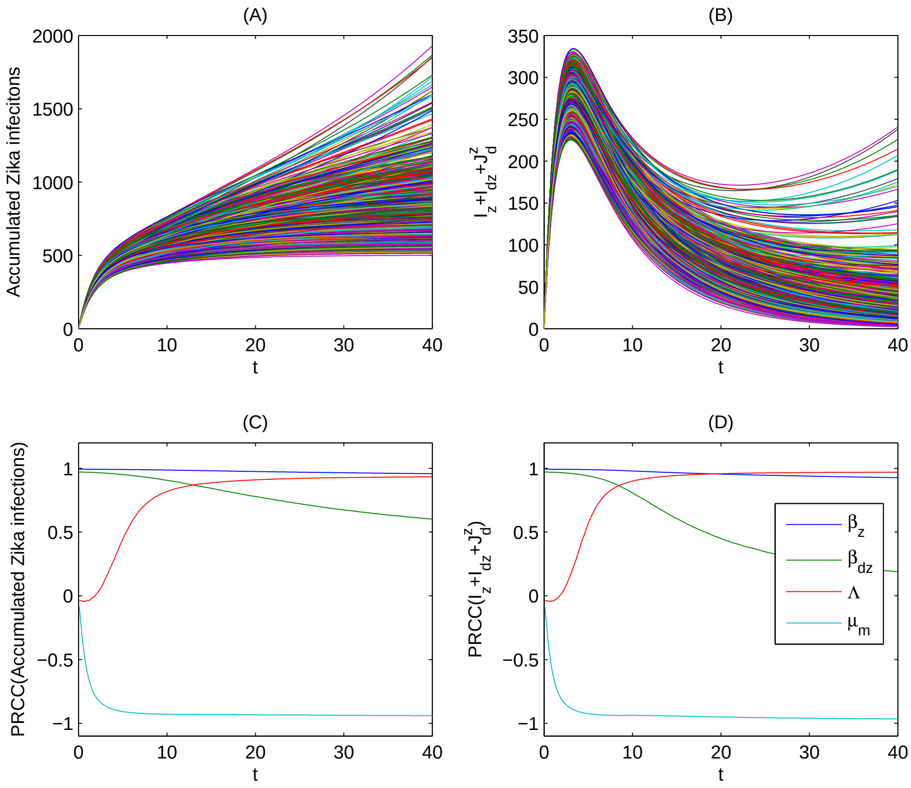

Figure S1: Sensitive analysis. (A-B) are plots of outputs of 1000 runs for the accumulated infections of Zika and the number of Zika infections, respectively. (C-D) are PRCC of the parameters β_z, β_{dz}, Λ and μ_m for (C) the accumulated infections of Zika and for (D) the daily number of Zika infections.

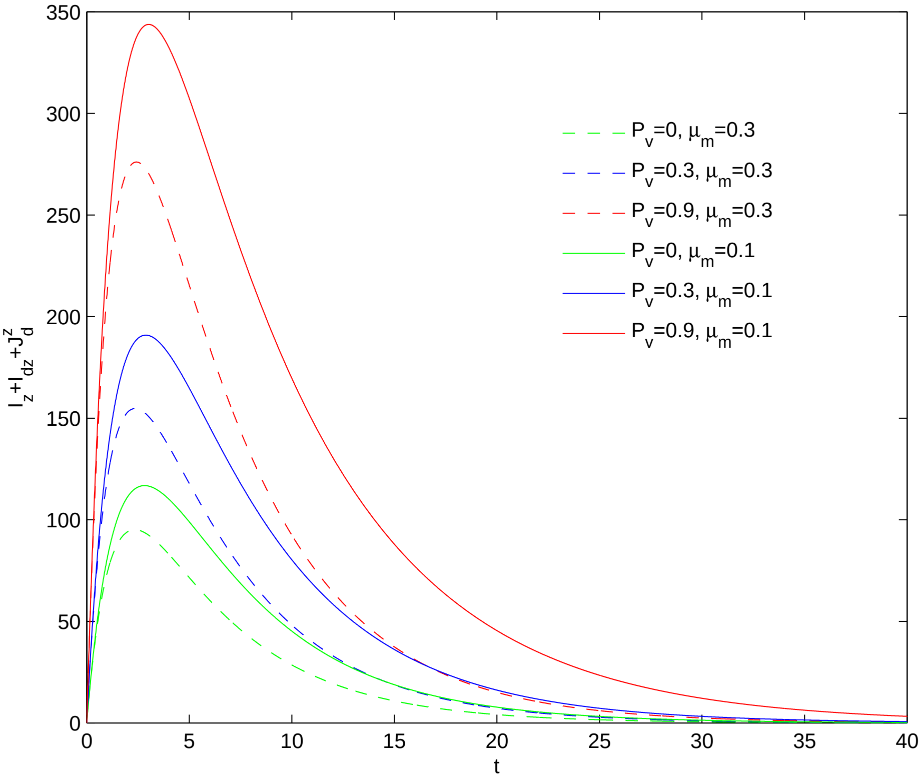

Figure S2: Plots of $I_z+I_{dz}+J_d^z$ as a function of time by varying the mosquito mortality rate μ_m. The other parameters are fixed as the same as those in Fig.7(A).
